# The complete chloroplast genome of *Edgeworthia chrysantha* (Thymelaeaceae)

**DOI:** 10.1080/23802359.2020.1832600

**Published:** 2020-11-03

**Authors:** Shiou Yih Lee, Kewang Xu, Wenbo Liao, Qiang Fan

**Affiliations:** a State Key Laboratory of Biocontrol and Guangdong Provincial Key Laboratory of Plant Resources, School of Life Science, Sun Yat-sen University, Guangzhou, China; b Co-Innovation Center for Sustainable Forestry in Southern China, College of Biology and the Environment, Key Laboratory of State Forestry and Grassland Administration on Subtropical Forest Biodiversity Conservation, Nanjing Forestry University, Nanjing, China

**Keywords:** Daphneae, genomic resources, illumina sequencing, traditional medicine, phylogenomics

## Abstract

*Edgeworthia chrysantha* is a subtropical plant with significant medicinal value. Herein, we assembled and characterized the complete chloroplast genome of *E. chrysantha* as genomic resource for future study. The genome consisted a total length of 172,446 bp, comprising of a large single-copy (LSC) region of 85,527 bp, a small single-copy (SSC) region of 2871 bp, separated by two inverted repeat (IR) regions of 42,024 bp each; a total of 139 genes were predicted for the chloroplast genome. Phylogenetic analysis showed that *E. chrysantha* is placed at the base of the Daphne group of tribe Daphneae, within the family Thymelaeaceae.

*Edgeworthia chrysantha* Lindl., from the family Thymelaeaceae, is naturally distributed in the eastern Asian region. The traditional usage of *E. chrysantha* stem includes as raw material for paper-making, while its flowers, barks, and roots are used as folk medicine due to its anti-bacterial properties, detumescence and acesodyne effects (Xu and Cai 2011). Due to its medicinal value, studies that focus on its phytochemical properties and bioactivities often overshadow the genetic information of this species. In this study, we aimed to characterize the complete chloroplast (cp) genome sequence of *E. chrysantha* to serve as a valuable genomic resource for this important medicinal plant species.

Total genomic DNA was extracted using DN15 Plant DNA Mini Kit (Aidlab Biotechnologies, China), according to the manufacturer’s protocol, from fresh leaves of *E. chrysantha* planted in the Germplasm Resource Nursery of Ornamental Plants, Guangzhou Institute of Forestry and Landscape Architecture, Guangdong province of China (N113°20′25″, E23°13′47″). Additional dried leaf specimens were kept in the Herbarium of SYSU (SYS) under the collection number LSY-THY-5001. A genomic library consisting of an insert size of 300 bp was constructed using TruSeq DNA Sample Prep Kit (Illumina, USA) and sequencing was conducted on an Illumina Novaseq platform. Approximately 6 Gb of raw data of 150-bp paired-ends reads were generated and genome assembly was conducted using NOVOPlasty (Dierckxsens et al. 2017). The *rbc*L sequence of *E. chrysantha* (Genbank accession number: MF349726) was selected as the seed sequence. Gene annotation was conducted using GeSeq (Tillich et al. 2017) and manually corrected.

The complete cp genome sequence of *E. chrysantha* (GenBank accession number: MT135125) was 172,446 bp in length, including a large single-copy (LSC) region of 85,527 bp, a small single-copy (SSC) region of 2871 bp, separated by a pair of inverted repeat (IR) regions of 42,024 bp each. A total of 139 genes were predicted, consisting of 93 protein-coding genes, 38 tRNA genes, and 8 rRNA genes. The overall GC content was 36.6%.

To understand the relationship of *E. chrysantha* within the family Thymelaeaceae, nine complete cp genome sequences from the family Thymelaeaceae were included in a phylogenetic analysis. The sequences were aligned using MAFFT (Katoh and Standley 2013), and a maximum-likelihood (ML) tree was constructed using RAxML (Stamatakis 2014). Two species, *Gossypium hirsutum* (Malvaceae) and *Eucalyptus grandis* (Myrtaceae), were included as outgroups. The ML tree showed that *E. chrysantha* is placed at the base of the Daphne group (including *Daphne*, *Stellera*, and *Wikstroemia*) with strong bootstrap support (Figure 1).

**Figure 1.**
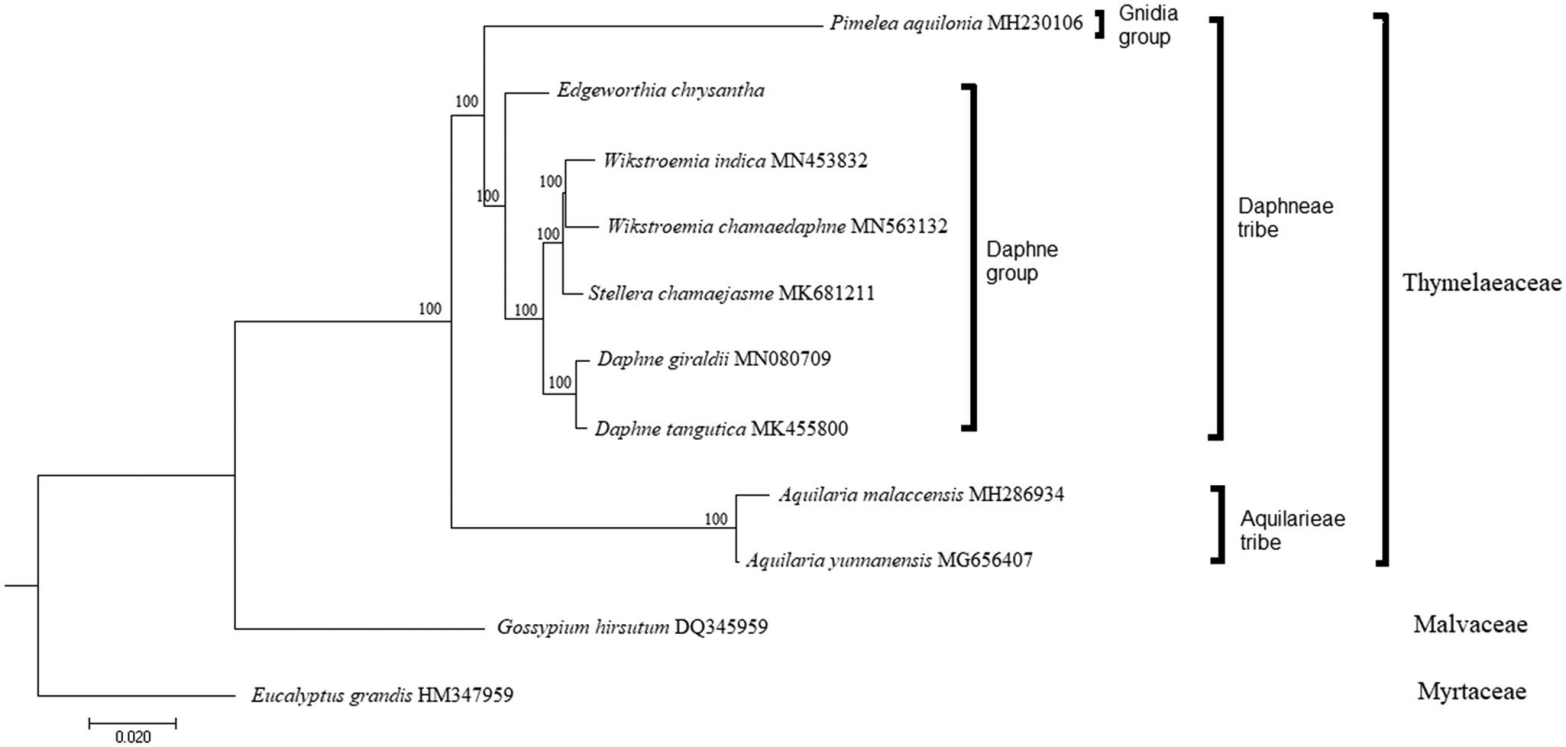
Maximum likelihood tree constructed based on the complete chloroplast genome sequences of nine species from the family Thymelaeaceae, with *Gossypium hirsutum* (Malvaceae) and *Eucalyptus grandis* (Myrtaceae) as outgroup. All branch nodes are indicated with bootstrap support values based on 1000 replicates.

## Data Availability

The data that support the findings of this study are openly available in the NCBI GenBank at http://www.ncbi.nlm.nih.gov, accession number MT135125.
